# Distant Metastases of an Atypical Meningioma 14 Years Later After Primary Tumor Resection: A Case Report and Review of the Literature

**DOI:** 10.1155/crip/5717203

**Published:** 2026-02-25

**Authors:** Kassaye Firde, Douglas Flieder, Maria Gonzalez, Laura Barry, Alessandro Bombonati, Israh Akhtar, Ibrahim Khalifeh, Curtis Miyamoto, Yuan Rong

**Affiliations:** ^1^ Department of Pathology and Laboratory Medicine, Temple University Hospital, Philadelphia, Pennsylvania, USA, temple.edu; ^2^ Department of Pathology and Laboratory Medicine, Fox Chase Cancer Center, Philadelphia, Pennsylvania, USA, fccc.edu; ^3^ Department of Pathology, UT Southwestern University Medical Center, Dallas, Texas, USA; ^4^ Radiation Oncology, Temple University Hospital, Philadelphia, Pennsylvania, USA, temple.edu

**Keywords:** extracranial metastases, meningioma, radiation therapy

## Abstract

Meningioma, one of the most common neoplasms of the central nervous system in adults, is most likely derived from the meningothelial cells of the arachnoid matter. Although most meningiomas have a benign clinical course and a relatively good prognosis, some can demonstrate more aggressive biological features, including local invasion and distant metastases. Extracranial meningioma metastases are rare, occurring in less than 1% of cases. We present a case of atypical meningioma status post resection with intracranial recurrence followed by multiple radiation therapies who developed extracranial metastases 14 years after primary tumor resection.

## 1. Introduction

Meningiomas are the most common primary central nervous system (CNS) tumors. Traditionally, meningiomas are classified into three WHO grades mainly based on morphological features: benign meningioma (WHO Grade 1), atypical meningioma (WHO Grade 2), and malignant meningioma (WHO Grade 3). Some significant changes for meningioma classification with the introduction of molecular biomarkers have been made according to the recent fifth edition of the WHO classification of CNS tumors and cIMPACT‐NOW Update 8 [1, 2]. CDKN2A/CDKN2B homozygous deletion and/or TERT promoter oncogenic variants are introduced as independent criteria for WHO Grade 3 meningiomas with Grade 1 or Grade 2 morphology. Brain invasion has been considered an independent morphological feature for the diagnosis of WHO Grade 2 meningioma. According to the new update, brain‐invasive but otherwise morphologically benign (BIOB) meningiomas should not be graded before molecular data is available. The other major update is that histologically low‐grade or borderline meningiomas with 1p deletion and concurrent 22q deletion/NF22 oncogenic variant should be classified as WHO Grade 2 if no Grade 3 molecular markers are present. For meningiomas with borderline morphological grading criteria, such as suspicious for brain invasion, or less than 3 out of 5 morphological features (high cellularity, clustered small cells with high N:C ratio, prominent nucleoli, sheet‐like growth pattern, tumor necrosis) for WHO Grade 2 meningioma, molecular testing is strongly recommended for final definite grading. In addition, meningiomas with papillary and rhabdoid features have been traditionally classified as WHO Grade 3. In the fifth edition of the WHO classification of CNS tumors, it is recommended that focal papillary architecture or rhabdoid feature alone in the absence of any other features of higher grade is not sufficient for designating tumors as CNS WHO Grade 2 or 3. For such cases, molecular testing is also recommended for the final grading. For all the meningiomas, the incidence of extracranial metastases is less than 1%, with a higher incidence in atypical meningioma (2%) and malignant meningioma (9%) [3]. Here, we report a patient who developed extracranial metastases 14 years after primary atypical meningioma resection followed by intracranial recurrence with multiple radiation therapies.

## 2. Case Presentation

A 68‐year‐old male with a history of parasagittal atypical meningioma (WHO Grade 2) status post gross total resection in 2010 received three times of Gamma Knife treatments in 2012, 2013, and 2021 for intracranial recurrence. In May 2024, the patient presented with 2 months of abdominal pain, early satiety, and unintentional weight loss. Abdominal CT scan demonstrated a 21 × 18 × 14 cm left upper quadrant abdominal mass along the gastric fundus. The mass showed internal calcifications and areas of necrosis, clinically suspicious for gastrointestinal stromal tumor (GIST). Suspected hepatic and pulmonary metastatic lesions with multiple enlarged upper abdominal and porta hepatic lymph nodes were also noted. A core biopsy of the gastric mass demonstrates a meningothelial neoplasm with increased cellularity, tumor necrosis, prominent nucleoli, and scattered mitotic figures (up to 4 mitoses/2 mm^2^). Whorls and pseudointranuclear inclusions were also noted. By immunohistochemistry, the lesional cells were positive for cytokeratin AE1/3, Cam5.2, EMA (focal), vimentin, D2‐40, E‐cadherin, and somatostatin receptor 2a (SSTR2a), Ki‐67 (clone MIB‐1) proliferation index up to 10%, but negative for progesterone receptor (PR) and GIST markers including CD117 (c‐kit) and DOG‐1 (Figure 1). Tumor cells were negative for other primary site markers including lung (TTF‐1), liver (HepPar‐1, arginase), prostate (NKX3.1), genitourinary (PAX8, Gata3), and pancreatobiliary tract (CK19, CDX2). Review of the concurrent cytopathology specimen and the previous primary atypical meningioma specimen demonstrated a similar morphology and immunoprofile. A liver biopsy was also performed, and the diagnosis was consistent with metastatic meningioma. Brain MRI in July 2024 showed tumor progression in the posterior parasagittal region. The patient received everolimus chemotherapy for extracranial metastases without radiation therapy in September 2024. Unfortunately, the patient passed away in December 2024 due to the disease progression.

Figure 1Histological and immunohistochemical features of the metastatic meningioma involving the stomach. (a) Gastric biopsy specimen. Demonstrate characteristic meningothelial cell features with increased cellularity, vaguely whorling structures, and occasional intranuclear pseudoinclusions. Hematoxylin and eosin stain, 400x. (b) Gastric biopsy specimen. Demonstrates focal tumor necrosis. Hematoxylin and eosin stain, 400x. (c, d) Concurrent cytopathology specimen. Demonstrates classic meningothelial cell features with occasional intranuclear pseudoinclusions and vaguely whorling structures (c). Some of the tumor cells with vacuolated cytoplasm (d). Hematoxylin and eosin stain, 400x. (e) Cytokeratin AE1/3 immunohistochemistry of the biopsy specimen. Tumor cells are positive for CK AE1/3, 100x. (f) Somatostatin receptor 2a (SSTR2a) immunohistochemistry of the biopsy specimen. Tumor cells are positive for SSTR2a, 200x.

**Figure:**
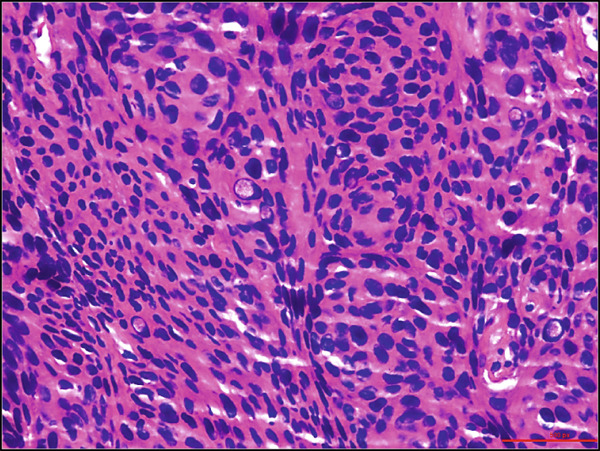
(a)

**Figure:**
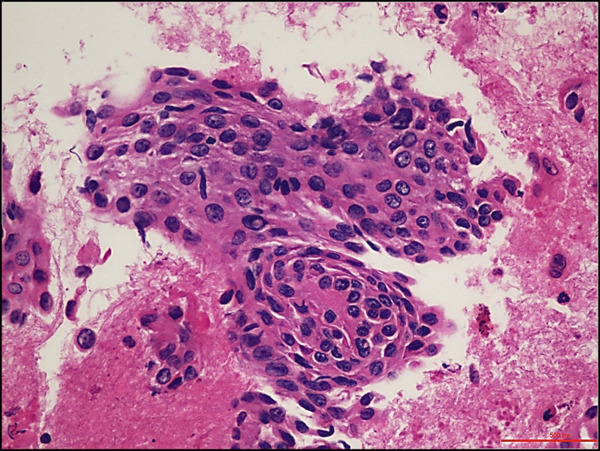
(b)

**Figure:**
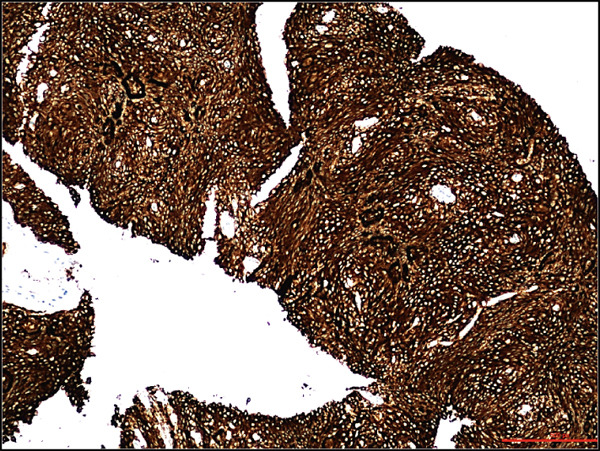
(c)

**Figure:**
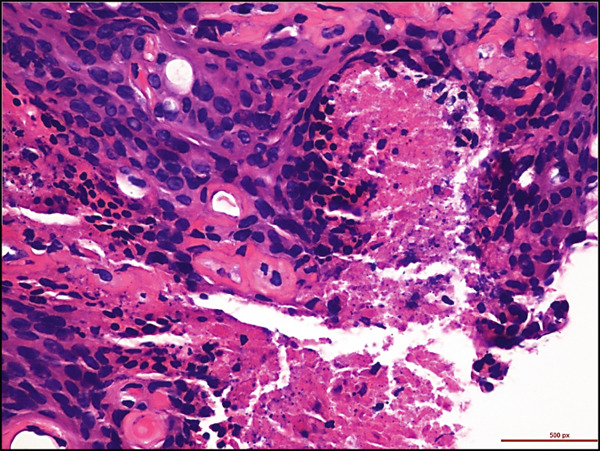
(d)

**Figure:**
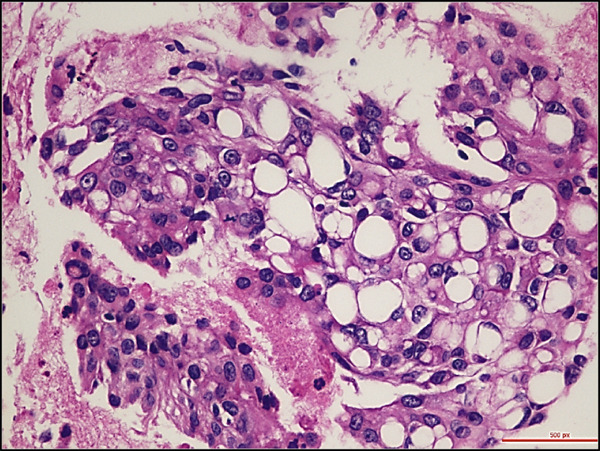
(e)

**Figure:**
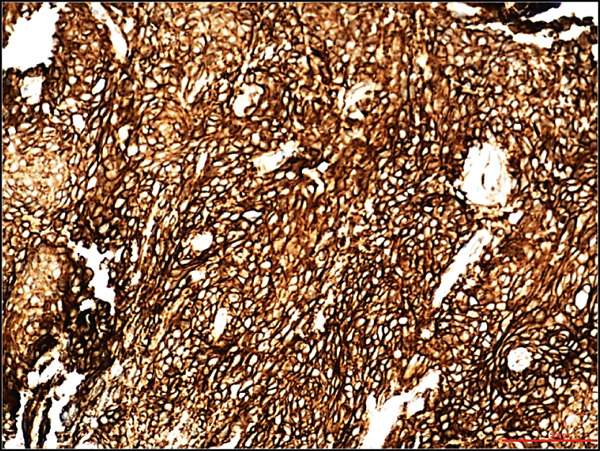
(f)

## 3. Discussion

Distant extracranial metastases of meningioma have been considered to be uncommon and mainly occur in malignant meningiomas (WHO Grade 3) [4, 5]. The incidence of metastasis in all meningiomas reported in most literature reviews is less than 1%. No definite criteria have been established to predict the ability of meningiomas to recur or metastasize. Several factors, including invasion into the venous sinus, intracranial recurrence, high‐grade histology, and papillary morphology, have been suggested to correlate with a higher risk for recurrence and/or distant metastasis [6]. Among these factors, the WHO histological grading is believed to be the most important factor in predicting recurrence or metastases. Malignant meningioma (WHO Grade 3) can have an incidence of metastasis ranging from 9% to 43%, according to the literature [3, 5]. One review also reported up to 56% of metastatic meningiomas occurred in benign (WHO Grade 1) and atypical meningiomas (WHO Grade 2) [7]. This finding indicates that other unknown factors independent of WHO histological grading may also be associated with distant metastases. Our case had an initial diagnosis of atypical meningioma with multiple intracranial recurrences even after radiation therapy. In order to better understand this aggressive behavior, DNA and RNA next‐generation sequencing (NGS) was performed on the primary atypical meningioma. NF2, CDKN2C, and NOTCH3 mutations were detected. However, no CDKN2A/CDKN2B and TERT promoter mutations were detected. Perivascular NOTCH3+ stem cells have been reported to be responsible for meningioma tumorigenesis and resistance to radiation therapy [8]. The most common sites of distant metastasis include the lung (60%) followed by the liver (34%), lymph nodes (18%), bones, pelvis and skull (11%), pleura (9%), vertebrae (7%), and mediastinum (5%) [6, 7, 9]. Metastases in abdominal organs, including the gastrointestinal tract, are also reported [10, 11]. Distant metastases often occur after the resection of the primary tumor, with a mean interval ranging from 58 to 76 months to detection of the first metastasis [6, 7]. The longest interval reported in the literature is 24 years [10]. Our case developed distant metastases 14 years later involving the lung, liver, and stomach. Initially, GIST was suspected clinically. We believe this is the first case of biopsy‐proven meningioma with gastric metastasis. The previously reported gastrointestinal metastatic meningioma involved duodenum [11]. For patients with a history of meningioma, especially with multiple intracranial recurrences after surgical resection, a possible extracranial metastatic disease should be included in the differential diagnosis in the situation of suspected systemic lesions [12]. The consensus on how to treat metastatic meningiomas has not been well established. Discovering specific molecular markers may provide a more personalized treatment option.

## Funding

No funding was received for this manuscript.

## Consent

No written consent has been obtained from the patient as there is no patient identifiable data included in this case report.

## Conflicts of Interest

The authors declare no conflicts of interest.

## Data Availability

The data that support the findings of this study are available on request from the corresponding author. The data are not publicly available due to privacy or ethical restrictions.
